# Morphological Study of the Newly Designed Cementless Femoral Stem

**DOI:** 10.1155/2014/692328

**Published:** 2014-06-16

**Authors:** Mohd Yusof Baharuddin, Sh-Hussain Salleh, Ahmad Hafiz Zulkifly, Muhammad Hisyam Lee, Alias Mohd Noor

**Affiliations:** ^1^Department of Biomedical Engineering, Faculty of Engineering, University of Malaya, 50603 Lembah Pantai, Kuala Lumpur, Malaysia; ^2^Centre for Biomedical Engineering Transportation Research Alliance, Universiti Teknologi Malaysia, 81310 Skudai, Johor, Malaysia; ^3^Department of Orthopaedic, Traumatology & Rehabilitation, Kuliyyah of Medicine, International Islamic University Malaysia, 25200 Kuantan, Pahang, Malaysia; ^4^Department of Mathematical Sciences, Faculty of Science, Universiti Teknologi Malaysia, 81310 Skudai, Johor, Malaysia

## Abstract

A morphology study was essential to the development of the cementless femoral stem because accurate dimensions for both the periosteal and endosteal canal ensure primary fixation stability for the stem, bone interface, and prevent stress shielding at the calcar region. This paper focused on a three-dimensional femoral model for Asian patients that applied preoperative planning and femoral stem design. We measured various femoral parameters such as the femoral head offset, collodiaphyseal angle, bowing angle, anteversion, and medullary canal diameters from the osteotomy level to 150 mm below the osteotomy level to determine the position of the isthmus. Other indices and ratios for the endosteal canal, metaphyseal, and flares were computed and examined. The results showed that Asian femurs are smaller than Western femurs, except in the metaphyseal region. The canal flare index (CFI) was poorly correlated (*r* < 0.50) to the metaphyseal canal flare index (MCFI), but correlated well (*r* = 0.66) with the corticomedullary index (CMI). The diversity of the femoral size, particularly in the metaphyseal region, allows for proper femoral stem design for Asian patients, improves osseointegration, and prolongs the life of the implant.

## 1. Introduction

Total hip arthroplasty (THA) has become common procedure for orthopaedic surgeons and it is used to restore the function of the hip joint lost to degenerative bone diseases such as osteoarthritis and rheumatoid arthritis. The morphology of the proximal femora is essential during preoperative planning because an accurate measurement helps the surgeon to select the best implant, which ensures long term success rates [1, 2]. Conventional methods use two-dimensional femur images from standard radiograph even though standard radiograph is imprecise compared to other medical imaging modalities such as computed tomography scanning and magnetic resonance imaging [3, 4]. However, computed tomography images have several disadvantages for implant design such as a slice thickness of 2–5 mm, slice spacing up to 10 mm, and errors in the real measurements of femur viewed in two dimensions [5–7]. Newer methods using three-dimensional femur models provided better information, which aids the surgeon in choosing the best implant design to fit with the anatomical features of the patient [4, 7–10].

Minimal studies of Asian femur morphology intricate the design process of the THA for this region [11–13]. Several studies regarding proximal femur morphology demonstrates the variations in size between Western and Asian femurs [8, 14, 15]. The Asian population is generally smaller in build and stature, but commercial off-the-shelf femoral stems currently available are manufactured in accordance with Western standards [11, 13, 16]. Biological fixation can be achieved by comprehending the morphology of the proximal femur prior design to achieve an optimum fit, fill implant, and promote bone ingrowth. Several studies in Japan emphasized obtaining precise information about the endosteal canal in a proximal femur, which is essential for metaphyseal support for cementless hip stems [17–21]. These researchers developed the cementless femoral stem that was specifically designed for dysplastic hips with proximal fitting anterolaterally flared stems, which has showed promising results. The primary stability enhanced bone ingrowths which prevent micromotion, loosening, and concomitant failure. We believed that the small physique of Asian patients has a peculiar femoral morphology that differs from Western populations. The objectives of this study were: (i) to provide a morphological description of the three-dimensional femoral model for Asian populations, and (ii) to analyze and correlate the ratios and indices regarding the endosteal canal, metaphyseal, and flares, which can be used as the guidelines for femoral stem design as well as in clinical practice.

## 2. Materials and Methods

**Figure 1:**
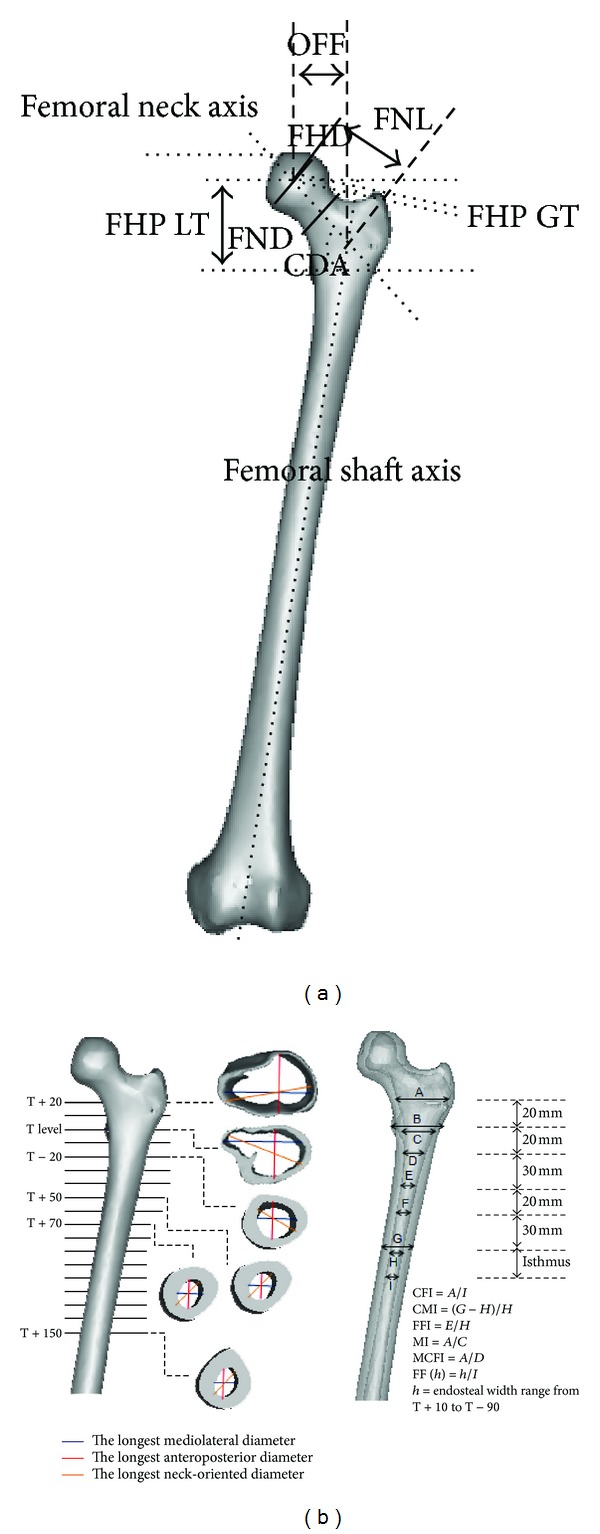
Morphology of the three-dimensional femora model (a) periosteal and (b) endosteal canal.

This prospective, cross-sectional study was carried out from January 2009 to December 2009 after obtaining approval from the National Medical Research Register (NMRR) and the local hospital ethics committee. We performed morphological studies of proximal femoral on 60 healthy femora (30 males and 30 females). The average age for all subjects was 25.01 ± 5.18 years. The average weight was 70.76 ± 14.38 kg for male and 53.31 ± 13.11 kg for female. The average height was 170.96 ± 6.37 cm for male and 156.02 ± 6.17 cm for female. Subjects were excluded from this study if they were pregnant, experienced prior femoral injuries or bone disease (osteoporosis, osteoarthritis), had an abnormal body mass index (BMI), had implants, or had received a computed tomography scan less than 6 months from the date their consent was filed. Computed tomography (CT) scans were performed using a four-row multislices CT scanner (Somatom, Volume Zoom, Siemens) and were conducted using 120 kV and 90 mAs. Other scanning parameters were set to 1.5 mm of recon increment, 1.25 mm of collimation, and 12.0 mm table feed per rotation. During the scan, the subjects were in a supine position with their feet stabilized using a specially designed wood jig to standardize the position of their feet. The proximal femoral images obtained were 3.0 mm thick with a resolution of 512 × 512 pixels. Gonad shields were used and no contrast media was administered. The CT scan images in DICOM format were imported into Mimics 10.0 software (Materialise, Leuvan, Belgium). The CT image threshold in Hounsfield units (HU) was classified to demarcate boundary regions between the cortical bone and the cancellous bone through profile line checking across the CT gray slice section. The threshold profile was set to 662–1988 HU for cortical bone and 148–661 HU for cancellous bone. The femora mask was converted into a three-dimensional model and then converted into a stereo lithography (STL) model and orthogonally cut into sections after measuring 10 mm intervals from the center of the lesser trochanter, T, as shown in Figure 1. The three-dimensional sliced femora were converted into stereo lithography models so that they could be accurately measured by commercial CAD software (Solidworks 2009 SP2.1, Dassault System, Massachusetts, USA). The following geometry parameter definitions for periosteal femurs were used to design femoral stems.

**Figure 2:**
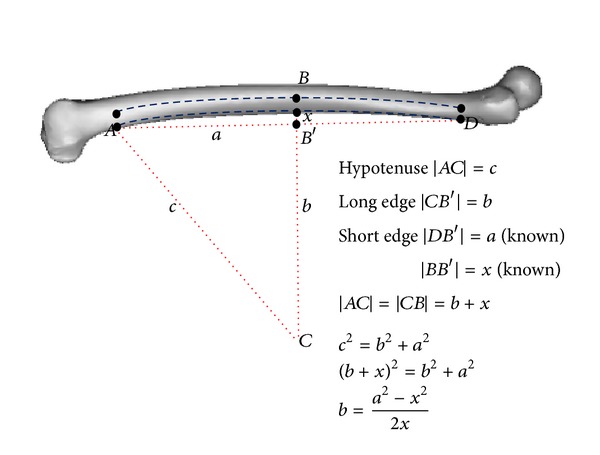
The measurement of anterior bowing from lateral view.

- Collodiaphyseal angle (CDA): angle between the femoral neck axis and femoral shaft axis.
- Femoral head offset (OFF): perpendicular distance between the femoral shaft axis and the femoral head center.
- Femoral neck length (FNL): distance between the femoral head center and the intersection point of the femoral shaft axis and femoral neck axis.
- Femoral head diameter (FHD): maximum diameter of the femoral head.
- Femoral neck diameter (FND): minimum diameter of the femoral neck.
- Femoral head position GT (FHP GT): vertical distance between the femoral head center and perpendicular line of femoral shaft axis.
- Femoral head position LT (FHP LT): vertical distance between the femoral head center and center of lesser trochanter, also known as the vertical offset.
- Anteversion: angle between the femoral neck axis and the line connecting two posterior condyles in transverse view [8, 15, 22].
- Anterior bowing: the imaginary arch's radius (*AC*) of the femoral curvature on lateral views as shown in Figure 2. Three points were determined on the posterior cortical arch: proximal and lower rim of lesser trochanter (*D*), midpoint (*B*′), and distal and condyle enlarged beginnings (*A*) [23–25].
- Bowing angle: angle between the central line of the proximal femoral diaphysis and the central line of the distal femoral diaphysis view [8, 15, 22].

Several authors found medullary canal diameters using a three-dimensional femora model that was more accurate than models based on conventional radiograph [4, 8–10, 26, 27]. The longest mediolateral, anteroposterior, and oblique medullary canal diameters were measured for each slice. We noted the existence of septum in all subjects from zero level, T to the osteotomy level slice, which narrowed the medullary canal. This septum partially separated the endosteal canal from the spongy bone of the lesser trochanter. The smallest endosteal canal in mediolateral directions was computed and considered to be the isthmus level. Several ratios and indices were computed to determine the correlation of the endosteal as demonstrated in Figure 1. The canal flare index (CFI) was calculated as the ratio of the endosteal diameter at the osteotomy level (T + 20) and the isthmus diameter in mediolateral directions [8]. Comparison of the CFI in our study and other populations was categorized as stovepipe (CFI < 3.0), normal (3.0 < CFI < 4.7), and champagne-flute (CFI > 4.7) shape [4, 8–10, 28, 29]. CFI in anteroposterior and neck-oriented (oblique) directions was measured similarly as in the mediolateral direction. These values indicated the endosteal canal opening from the osteotomy level slice to the isthmus [10]. In addition, the femora medullary canal enlargement rate from these levels can be determined by subtracting the endosteal diameter from the top to the bottom level [10]. To better understand the femora flare, we calculated the ratios within these two levels (T + 10 until T − 90) with an endosteal diameter of 100 mm below zero level, T in the mediolateral direction [30].

The metaphyseal cavity was essential for providing proximal fixation support especially for the cementless femoral stem. The metaphyseal canal flare index (MCFI) was computed as the ratio of the endosteal diameter 20 mm above and 20 mm below the center of lesser trochanter in mediolateral, anteroposterior, and oblique directions [10]. The correlation between MCFI and CFI was determined to be statistically significant. Next, the metaphyseal index (MI) was calculated as the ratio of the endosteal diameter at the osteotomy level and zero level, T [28]. The corticomedullary index (CMI) was used as the indicator for deciding if the hip replacement should be cemented or cementless for centered femurs [30]. The CMI was computed as the ratio of the cortical thickness (lateral and medial) and endosteal diameter at 100 mm below zero level, T, as illustrated in Figure 1. The correlation between the CMI and femoral flare index (FFI) was determined and used to select a standard or custom made implant [30]. The FFI was computed similar to the CFI in the mediolateral directions.

The measurements data was statistically analyzed using SAS 4.3 software (SAS Institute Inc., Cary, NC, USA). The value *P* < 0.05 was set to determine whether the data was statistically significantly different between genders. Normality assumption for each group of data was verified using the Cramer-von Mises method. The folded *F* method was used to examine the equality of data variances when the data was normally distributed. Probability was then checked using a *t*-test (either the Pooled or Satterthwaite method) depending on the equality of the variance. The Pearson correlation coefficient was used to measure the correlation between medullary canals and the endosteal ratios or indices.

## 3. Results

The comparison of periosteal femoral in different populations is depicted in Table 1. We compared our data to data from Thai [15], Indian [22], Nepalese [13], Caucasian [8], Turkish [29], and Swiss [4] populations. The collodiaphyseal angle for Malays was higher (130.46°) compared to other populations except Nepalese populations. However, due to the small physique of the Malay population, smaller sizes had been anticipated in several parameters such as femoral head offset, femoral neck length, and femoral head diameter. There was a 16.65 mm difference between Malay and Swiss populations in terms of femoral head offset, which is a crucial parameter for determining the size of the hip stem during preoperative planning. The anteversion and bowing angle for Malay populations were also different.

**Figure 3:**
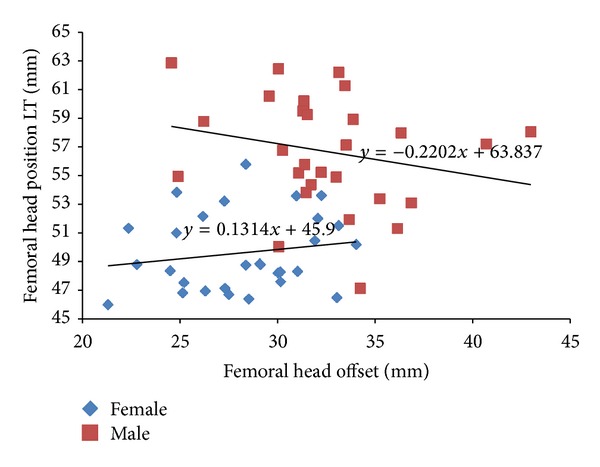
The morphological relationship between femoral head position from lesser trochanter and femoral head offset based on gender.

The canal flare index (CFI) used in this study classified most of the samples as having a normal shape, which was in the range of 3.0–4.7 [8]. The morphological relation between the femoral head position from the lesser trochanter and the femoral head offset is shown in Figure 3. The femoral head position for males was in the range of 47.11–62.84 mm and for females it was in range of 45.98–55.79 mm. The femoral head offset for males was in the range of 24.56–42.99 mm and for females it was in the range of 21.31–34.03 mm. The equations for males and females are *y* = 63.83 − 0.220*x* and *y* = 45.9 + 0.131*x*, respectively. No correlation between femoral head position from the lesser trochanter and femoral head offset was found for either gender.

**Figure 4:**
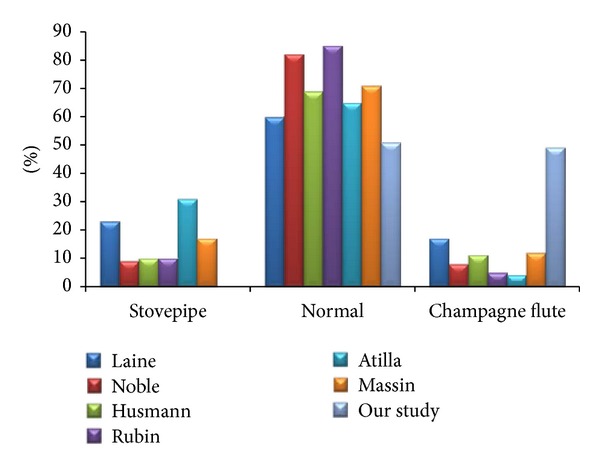
Histogram of the canal flare index (CFI) between our study and other populations. The CFI was categorized according to stovepipe (CFI < 3.0), normal (3.0 < CFI < 4.7), and champagne-flute (CFI > 4.7) shape.

The mean and standards deviations of medullary canal diameters for each gender in mediolateral (ML), anteroposterior (AP), and oblique (OB) directions at different levels are shown in Table 2. We compared our data with Finnish and Indian populations [10, 26]. The Finns had larger endosteal diameter values compared to the samples used in our study and Indian populations, except in the metaphyseal region where small differences could be seen. About 81.67% of Malay femora had an isthmus position that was from 100 to 120 mm below zero level, T. The canal flare index (CFI) in our study was 4.65 ± 0.83 which placed it in the normal category [8]. We compared the distribution of CFI from our study with previous studies from Finnish [10], Caucasian [8, 9], Swiss [4], Turkish [29], and French [28] populations as shown in Figure 4. Our study showed that 51.72% of samples were classified as having a normal shape and 48.28% as having a champagne-flute shape. No stovepipe shaped femora were found in our study but could be found in other populations.

**Figure 5:**
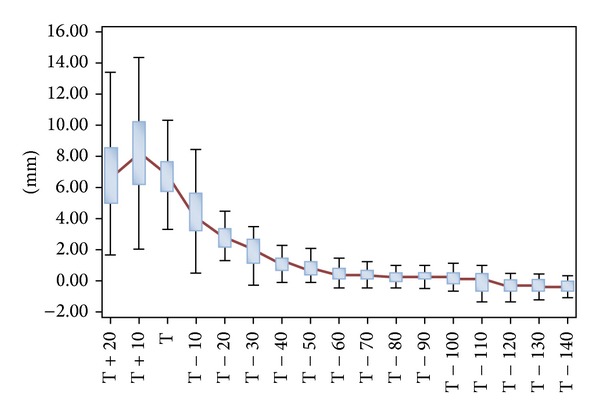
Femora medullary canal enlargement rate showed as a box plot. The red line connected the median for each difference in mediolateral endosteal diameter.

**Figure 6:**
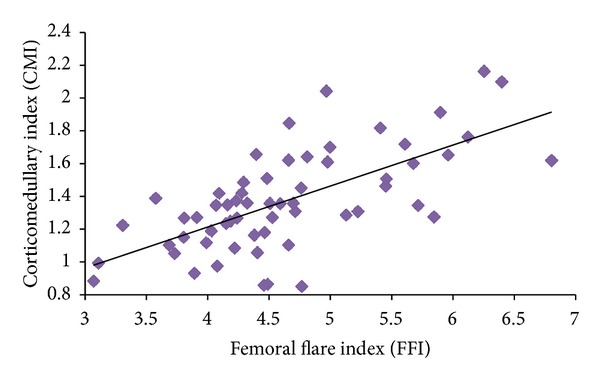
Correlation between the corticomedullary index (CMI) and femoral flare index (FFI).

The endosteal enlargement rate (interval 10 mm for each slice) had the highest value at the metaphyseal section up to 20 mm above zero level, T, as illustrated in Figure 5. The femora cavity enlargement gradually decreased at the diaphyseal region. There was a poor correlation (*r* = 0.14–0.48) between the CFI and MCFI as shown in Table 3. However, the CMI correlated very well (*r* = 0.66) with FFI as illustrated in Figure 6. The metaphyseal index (MI) was statistically significant (*P* = 0.1733) between females and males with 1.56 ± 0.21 and 1.49 ± 0.17, respectively.

## 4. Discussion

Available commercial hip prostheses are regarded as universally usable for all femora types thus neglecting the differences in hip joint morphology among populations [13]. The differences in Asian femoral morphology compared to Western populations are due to genetic, physique, and peculiar lifestyles [13]. Currently, global implant manufacturers are producing smaller sizes to cater to Asian patients, but not all requirements are met, especially for the medullary canal [12, 13, 31]. The implants are usually designed using Western databases and the linear and angular specifications varies [8, 13, 31, 32]. As a result of relying on Western standards, these implants and device are bigger in size and more bone stock in the medullary canal is put a risk during the surgery. Furthermore, other parameters that are generally used in determining the size of the implant are significantly different from the Western parameters such as the collodiaphyseal angle (CDA), femoral head offset (OFF), femoral head diameter (FHD), and the isthmus position and diameter. The comparison between the Caucasian and Hong Kong Chinese demonstrates higher CDA of 136° and 135°, respectively [14]. Manufacturers are inclined to produce smaller CDAs to increase the femoral head offset increasing soft tissue tension and reducing the probability of dislocation in THA [33, 34]. However, a declination of CDA of 1° will reduce cup anteversion by 2° and increase cup inclination by 0.45° [34].

Commercial femoral stems have a universal design of 135°, which differs from the femoral stems of the populations used in our study as well as other populations, as shown in Table 1. This design shortened the femoral head offset, which needs restoration by trochanteric osteotomy [28]. The femoral head offset is essential for improving hip stability, enhancing the range of motion (ROM) of the abduction, and ameliorating abductor strength [28, 35, 36]. A difference of 3.5 mm in femoral head offset was reported due to femoral anteversion when two-dimensional and three-dimensional methods of measurement were used [37]. The femoral anteversion can vary from 22° to 50° and can cause distortion of the femoral head offset, if conventional radiograph is used [9, 37]. Approximately 60% of cases showed that the femoral head offset was not restored when using 135° CDA and 32% of cases had the same result when using 131° CDA implants [33]. However, several studies indicated that there was no correlation between the femoral head offset and femoral anteversion because periosteal features were independently compared with the endosteal canal [37, 38]. After a femora neck is resected during surgery, it is crucial that the center of the femoral head is in the same position it was prior to the surgery to restore the offset and femoral length [37]. In general, femoral head position and the femoral head offset are chosen as anatomical landmarks because of their visibility during surgery and they are used to assess the success of the surgery [37]. There was no correlation found between these two parameters in this study, as illustrated in Figure 3. The femoral head position in this study (53.14 mm) was in the same range (48.94–59.10 mm) as other populations but our femoral head offset was relatively small (30.35 mm), as shown in Table 1. Several studies showed that the femoral head position from the greater trochanter was higher than the femoral head center between 8 and 9.5 mm [37–39]. Misconceptions about a similar perpendicular line with the femur axis should be avoided to prevent limb length discrepancy after THA [37, 39]. Other important parameters are the bowing angle and the anterior bowing, which is vital in prosthesis design [22]. Extreme bowing femora influence the stability of the implant especially with the cementless hip stem at distal diaphysis leading to overreaming, which risks more bone loss during surgery to prevent femora fracture [40, 41].

Three-dimensional images of femurs provided additional information about endosteal canal morphology. The diaphyseal region showed clear boundaries between the cancellous bone and cortex due to the thin transition zone [10]. However, the threshold needs to be clearly identified due to the difficulty of differentiating the cancellous bone from the cortex at the metaphyseal region [10, 42]. Comprehending the actual morphology of the metaphyseal region will lead to better stem fixation in the medullary canal [18, 20]. Furthermore, optimum fits and fills of the metaphyseal region sustain maximum loading to the femora [43]. Walker et al. indicated the significance of proximally fitting and considered that a femoral stem below the zero level was not essential for lateral flare cementless anatomical stems [44]. In addition, this type of stem will reproduce the compressive force between the femora head and greater trochanter [45].

Several studies used indices that can be used to understand the morphology of the metaphyseal region [8–10, 28]. Noble et al. suggested using the canal flare index (CFI) to classify the shape of the femora endosteal with a ratio between the osteotomy level slice and the isthmus [8]. Furthermore, Husmann et al. described more flare indices as the anatomical cementless femora guidelines with detail features on zone capital that optimized the hip prosthesis design and preoperative selection [9]. Laine et al. demonstrated that the metaphyseal canal flare index (MCFI) could be used to differentiate between the diversity of the proximal femora endosteal shapes [10]. In addition, Massin et al. pointed out the importance of the metaphyseal index (MI) for producing a series of mono block stem sizes that provided adequate fill in the frontal plane [28]. They presumed that cementless stem surgeries placed more emphasis on fitting than on filling [28].

**Figure 7:**
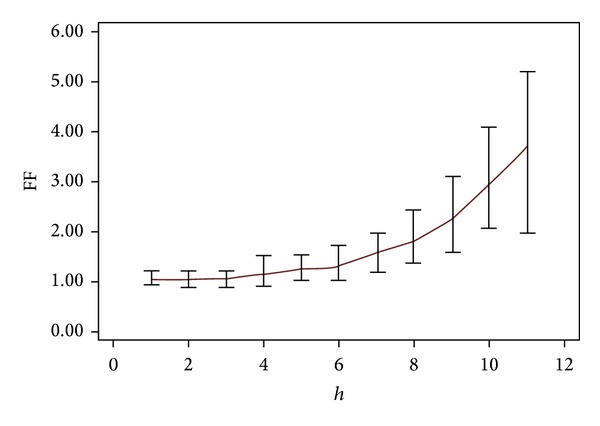
Femoral flare (FF) according to the height (*h*). FF was measured from level T + 10 until T − 90.

Conversely, the metaphyseal region plays an important role in assuring that the implant rests in the femoral canal. Our study revealed poor correlations between the CFI and MCFI as shown in Table 3. This indicated that the CFI cannot be used alone to represent the entire femora because the endosteal cavity opening varied from the metaphyseal to diaphyseal level. The medullary canal opening was greater at 10 mm above zero level, T compared with the osteotomy level. Fessy et al. used the flare ratio to describe the medullary canal opening due to the debatable methodology of the radiography data [30]. Our study showed a femoral flare of 3.71 at 10 mm above the zero level that gradually stabilized at the diaphyseal region as illustrated in Figure 7. Several studies in Japan considered these differences when they designed implants and focused on the metaphyseal region anterolaterally flared for loading purposes [17–20, 46]. Laine et al. emphasized the selection of the cementless stem from the metaphyseal region (MCFI) rather than the CFI [10]. They suggested that the variety of stem design was based on the metaphyseal region being established because a single design cannot be used for all femora shapes. Fessy et al. introduced a new method involving the corticomedullary index (CMI) and the femoral flare index (FFI) [30]. Our results were in accordance with Fessy et al. [30] indicating that there was a good correlation (*r* = 0.66) between CMI and FFI. Fessy et al. also suggested using cemented stems if the CMI < 1 and cementless stems if the CMI > 1 [30]. Furthermore, they proposed using a standard cementless stem if the FFI is within range of 3–4.5 and custom made cementless stems for any other cases [30].

Several studies introduced other indices and ratios that can be used to determine whether cemented or noncemented stems were the best option prior to surgery [8, 47, 48]. Noble et al. discussed the use of noncemented stems if the femur was categorized as having a champagne-flute shape (CFI > 4.7) [8]. In addition, Spotorno and Romagnoli proposed using cemented stems if the morphological cortical index (MCI) was less than 2.3 and noncemented stems if the MCI was more than 3 [48]. Dorr suggested cemented stems if the canal calcar ratio (CCR) was beyond 0.75 [47].

**Figure 8:**
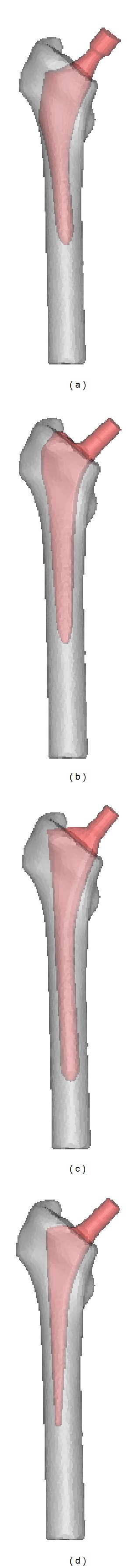
Commercial cementless femoral stem inside the femur (a) ABG, (b) Alloclassic, (c) AML, and (d) CLS.

One major limitation of this study was the comparative analysis with elder group from other populations. As far as the authors are aware, there is no literature report on hip morphology of the young age group. Currently, we continued collecting the data as these data were beneficial for future implant designs which integrating anatomical designs that may be applicable for larger number of populations in Asia. Furthermore, this reduced the fabrication cost for the cementless femoral stem and prevented the implant geometrical mismatch. Several commercial cementless femoral stems such as ABG, Alloclassic, AML, and CLS which were implanted within the three-dimensional femur models were showed in Figure 8. Nevertheless, further analysis such as finite element and biomechanical testing was required to validate this newly designed cementless femoral stem before clinically used.

## 5. Conclusion

We would like to emphasize the differences between Asian and Western femoral morphology and point out that this difference should be used as a guide to improve the design of commercially available femoral stems particularly for Asian populations. By comprehending the peculiar characteristic of the Asian femur, better designs with optimal fit and fill can be produced, which will prolong the lifetime of the implant and inhibit other complications such as micromotion, loosening, stress shielding, and fractures.

## Figures and Tables

**Table 1:** Comparison of the femoral periosteal morphology in different populations.

Parameters	Our study (Malay) (*n* = 60)	Mahaisavariya et al. [15] (Thai) (*n* = 108)	Rawal et al. [22] (Indian) (*n* = 98)	Mishra et al. [13] (Nepalese) (*n* = 50)	Noble et al. [8] (Caucasian) (*n* = 80)	Atilla et al. [29] (Turkish) (*n* = 114)	Rubin et al. [4] (Swiss) (*n* = 32)
Collodiaphyseal angle (°)	130.46 ± 4.02	128.04 ± 6.14	124.42 ± 5.49	132.60 ± 8.36	125.40	128.40 ± 4.75	122.90 ± 5.76
Femoral head offset (mm)	30.35 ± 4.26	—	40.23 ± 4.85	—	—	42.70 ± 6.54	47.00 ± 7.20
Femoral neck length (mm)	45.30 ± 4.74	46.22 ± 5.14	48.40 ± 5.66	—	—	—	—
Femoral head diameter (mm)	40.81 ± 3.43	43.98 ± 3.47	45.41 ± 3.66	44.26 ± 3.58	45.90	45.80 ± 4.17	43.40 ± 2.26
Femoral neck diameter (mm)	28.95 ± 3.37	—		34.42 ± 3.30	—	—	—
Femoral head position LT (mm)	53.14 ± 4.87	48.94 ± 4.95	52.33 ± 3.19	—	—	59.10 ± 7.74	56.10 ± 8.20
Femoral head position GT (mm)	5.29 ± 4.22	—	—	—	—	—	—
Anteversion (°)	19.10 ± 8.67	11.37 ± 7.65	10.90 ± 4.22	15.41 ± 5.21	10.00	—	—
Bowing angle (°)	2.28 ± 1.19	5.75 ± 1.37	8.15 ± 2.08	—	9.00	—	—
Anterior bowing (mm)	1123.72 ± 234.83	—	—	—	—	—	—
Canal flare index	4.65 ± 0.83	—	4.23 ± 2.97	—	—	—	3.36 ± 0.75

Data are presented as mean ± SD. LT: lesser trochanter.

**Table 2:** Comparison of the femoral endosteal canal morphology in different populations.

Level/index	Our study (Malay) (*n* = 60)			Laine et al. [10] (Finnish) (*n* = 50)			Sen et al. [26] (Indian) (*n* = 50)		
	ML	AP	OB	ML	AP	OB	ML	AP	OB
T + 20	44.05 ± 4.59	31.12 ± 3.70	46.21 ± 4.63	45.42 ± 4.46	31.39 ± 3.45	47.09 ± 4.98	—	—	—
T + 10	36.97 ± 4.85	27.67 ± 3.39	38.99 ± 4.81	35.49 ± 4.23	28.55 ± 3.06	37.53 ± 4.71	33.99 ± 6.34	26.41 ± 4.69	31.80 ± 5.93
T	29.30 ± 4.75	23.56 ± 3.08	38.16 ± 5.12	28.73 ± 3.21	25.58 ± 2.86	31.01 ± 3.65	27.27 ± 6.23	23.44 ± 3.78	29.21 ± 6.51
T − 10	22.69 ± 3.73	20.50 ± 3.03	30.31 ± 5.40	25.03 ± 2.58	23.85 ± 2.32	27.58 ± 2.53	21.10 ± 4.23	19.72 ± 3.63	22.54 ± 5.46
T − 20	18.45 ± 2.93	17.54 ± 2.93	20.78 ± 3.35	20.41 ± 2.14	20.71 ± 2.47	23.19 ± 2.74	17.92 ± 3.37	17.01 ± 3.01	18.71 ± 3.92
T − 30	15.55 ± 2.71	15.39 ± 3.01	17.62 ± 3.20	17.74 ± 1.93	18.27 ± 2.39	20.08 ± 2.52	15.32 ± 3.10	14.89 ± 2.78	16.00 ± 3.18
T − 40	13.54 ± 2.26	13.80 ± 2.69	15.89 ± 3.04	15.73 ± 1.97	16.77 ± 2.56	18.68 ± 2.51	12.40 ± 2.07	13.11 ± 2.39	13.11 ± 1.99
T − 50	12.51 ± 2.14	13.36 ± 2.57	15.11 ± 2.69	—	—	—	—	—	—
T − 60	11.63 ± 2.01	13.23 ± 2.67	14.52 ± 2.63	13.43 ± 2.12	15.92 ± 2.67	17.33 ± 2.65	11.50 ± 2.12	12.87 ± 2.51	12.33 ± 2.21
T − 70	11.08 ± 1.83	13.13 ± 2.56	14.50 ± 2.70	—	—	—	—	—	—
T − 80	10.74 ± 1.83	12.99 ± 2.56	14.12 ± 2.50	—	—	—	11.15 ± 2.08	12.93 ± 2.52	11.99 ± 1.95
Isthmus	9.73 ± 1.80	13.12 ± 2.46	13.90 ± 2.43	11.06 ± 1.88	14.09 ± 2.81	14.79 ± 2.96	—	—	—
T − 150	10.85 ± 1.53	13.65 ± 1.87	14.27 ± 1.84	12.65 ± 2.03	14.82 ± 2.64	15.59 ± 2.69	—	—	—

Data are presented as mean ± SD. T: lesser trochanter.

**Table 3:** Femoral flare indices and their correlations.

			Pearson correlation coefficients, *N* = 60 Prob > |*r*| under H0: Rho = 0					
			CFI			MCFI		
	Mean	SD	ML	NO	AP	ML	NO	AP
CFI								
Mediolateral	4.64	0.81	1.00	0.65	0.50	0.42	0.14	0.18
				<0.0001	<0.0001	0.0009	0.3025	0.1797
Neck-oriented	3.39	0.51	0.65	1.00	0.78	0.34	0.29	0.29
			<0.0001		<0.0001	0.0072	0.0241	0.0234
Anteroposterior	2.43	0.41	0.50	0.78	1.00	0.31	0.14	0.48
			<0.0001	<0.0001		0.0172	0.2776	<0.0001
MCFI								
Mediolateral	2.41	0.25	0.42	0.34	0.31	1.00	0.61	0.45
			0.0009	0.0072	0.0172		<0.0001	0.0004
Neck-oriented	2.25	0.23	0.14	0.29	0.14	0.61	1.00	0.57
			0.3025	0.0241	0.2776	<0.0001		<0.0001
Anteroposterior	1.80	0.21	0.18	0.29	0.48	0.45	0.57	1.00
			0.1797	0.0234	<0.0001	0.0004	<0.0001	

CFI: canal flare index; MCFI: metaphyseal canal flare index; SD: standard deviation; ML: mediolateral; AP: anteroposterior; and NO: neck-oriented.
